# h5vc: scalable nucleotide tallies with HDF5

**DOI:** 10.1093/bioinformatics/btu026

**Published:** 2014-01-21

**Authors:** Paul Theodor Pyl, Julian Gehring, Bernd Fischer, Wolfgang Huber

**Affiliations:** EMBL Heidelberg, Genome Biology Unit, Meyerhofstr. 1, 69117 Heidelberg, Germany

## Abstract

**Summary:** As applications of genome sequencing, including exomes and whole genomes, are expanding, there is a need for analysis tools that are scalable to large sets of samples and/or ultra-deep coverage. Many current tool chains are based on the widely used file formats BAM and VCF or VCF-derivatives. However, for some desirable analyses, data management with these formats creates substantial implementation overhead, and much time is spent parsing files and collating data. We observe that a *tally* data structure, i.e. the table of counts of nucleotides × samples × strands × genomic positions, provides a reasonable intermediate level of abstraction for many genomics analyses, including single nucleotide variant (SNV) and InDel calling, copy-number estimation and mutation spectrum analysis. Here we present h5vc, a data structure and associated software for managing tallies. The software contains functionality for creating tallies from BAM files, flexible and scalable data visualization, data quality assessment, computing statistics relevant to variant calling and other applications. Through the simplicity of its API, we envision making low-level analysis of large sets of genome sequencing data accessible to a wider range of researchers.

**Availability and implementation:** The package **h5vc** for the statistical environment R is available through the Bioconductor project. The HDF5 system is used as the core of our implementation.

**Contact:** pyl@embl.de or whuber@embl.de

**Supplementary information:** Supplementary data are available at *Bioinformatics* online.

## 1 MOTIVATION

There is interest in analyses of cancer genome data across large cohorts (Kandoth *et al.*, 2013), but the standard file formats are not well suited to the task. The BAM format (Li *et al.*, 2009) provides low-level information (alignments), but is resource-hungry, especially for data from many samples at high depth. On the other hand, the VCF format (Danecek *et al.*, 2011) provides high-level information and focuses on reporting positive variant calls, while reporting of negative calls is usually not attempted and can be expected to encounter scalability limitations. However, absence of evidence is not evidence of absence: just considering every position that is not mentioned in a VCF file a ‘no variant’ would imply a high false-negative rate, especially in the face of subclonality and uneven coverage.There is a need for an auxiliary format that is scalable, compact and accessible from multiple platforms.

## 2 HDF5

We use HDF5 (The HDF Group, 2010) as the core of our implementation. HDF5 is designed to store large arrays of numerical data efficiently, scales well with the size of the datasets, supports compression and is available on many platforms in the form of libraries for different programming languages including C/C++, Java, Python, Matlab and R.

Our implementation relies on the **rhdf5** Bioconductor package (Fischer and Pau, 2012) for low-level access functions to HDF5 files. We store the mismatch tally in a dataset called **Counts** and further quantities in the datasets **Coverages**, **Deletions** and **Reference**. The four datasets, which can be thought of as large arrays of integers, are defined as follows:

**Table:** 

**Counts:**	[bases × samples × strands × positions]
**Coverages:**	[samples × strands × positions]
**Deletions:**	[samples × strands × positions]
**Reference:**	[positions]

Within an HDF5 file, data are stored in a hierarchical structure consisting of groups and datasets. This layout is analogous to a file system where groups represent folders and datasets represent files. We use groups to represent the organizatorial units *cohort* and *chromosome*. In the filesystem analogy, the **Counts** dataset of e.g. chromosome *chr7* of cohort *ExampleCohort* will be stored at location/ExampleCohort/chr7/Counts in the HDF5 file (Supplementary Table S1 and Supplementary Fig. S1).

## 3 FEATURES

The tally file size is determined mainly by the genome size and the number of samples and not by the depth of coverage. By explicitly including the sample as a dimension of the data matrix, we can scale from single-sample or pairwise comparisons to cohort-level analyses involving thousands of samples without having to open thousands of file connections and parsing as many files. The use of R/Bioconductor (Gentleman *et al.*, 2004) for analyses and HDF5 for data storage provides platform independence and allows scientists to interact with their data on multiple operating systems. HDF5 tallies are small in comparison with BAM files, e.g. a dataset of 21 human exome sequencing samples used ∼150 GB of storage (at ∼100 million reads per sample), whereas the tally file took only 6.3 GB independent of the per-sample coverage. The tally can be interacted with through any of the languages that have HDF5 libraries (Section 2). Representing the mismatch tallies of a whole cohort within one array allows for convenient analyses across positions and samples. The central tool for interacting with HDF5 tally files is the h5dapply function provided in the **h5vc** package. It allows the user to specify a function that will be applied to the tally in a blockwise fashion along a specified dimension of the data. By default, blocks along the genomic position axis are used; in larger cohorts, applying functions in blocks along the sample axis becomes an interesting option. Blockwise processing allows for efficient use of available I/O, CPU and memory resources.

We note that nucleotide tallies do not store information on whether nearby events were seen on the same sequence fragment. Therefore, this data structure does not replace BAM files in applications, such as read-based phasing or the calling of large structural variations.

We provide two sets of tools for creating tally files (functions in the R package, and standalone Python scripts), documented in the vignettes *Creating Tallies with h5py/Python* and *Creating Tallies within R* of the **h5vc** package. Creating an HDF5 tally is an initial investment of time and compute resources that pays off through ease of use in downstream analyses. The Python script for creating tallies processed the 21 human exomes mentioned above at a rate of 1000–1700 reads per second. Processing a single chromosome and sample took between 5 min and 4 h (chrY vs. chr1), and a final merging step to collate datasets from all samples into one tally file took up to 35 h to complete when using compression.

## 4 GENOMIC ANALYSES WITH H5VC

The **h5vc** package provides basic functionality for many common genomic analyses, e.g. variant calling, visualization, quality control and mutation spectrum analysis, as well as a framework for implementing new algorithms easily.

### 4.1 Visualization

**Fig. 1.**
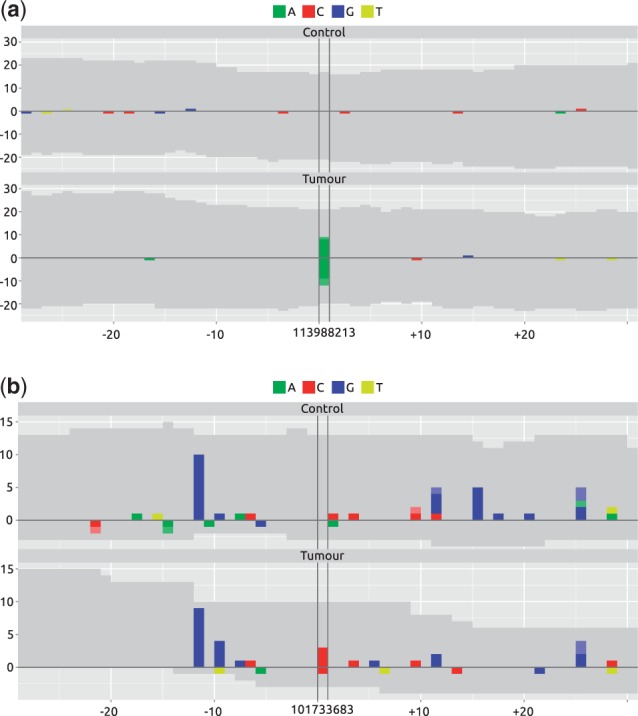
mismatchPlots of two candidate variant sites. Each sample (Control, Tumor) is shown in a separate panel, with the genomic position as a common *x*-axis centered around the position of the variant. Along the *y*-axis, alignment statistics of the forward and reverse strand are shown as positive and negative values, respectively. Gray areas represent coverage by sequences matching the reference, and colored areas represent mismatches, deletions and insertions. (**a**) Variant is present in the tumor sample but not in the control. (**b**) Variant of comparable position specific statistics as (a). Note the noisiness of the region, which is not immediately obvious from the position-specific values alone

An important part of variant calling is quality control. After automated procedures have reduced the number of potential variant sites to a manageable scale, rapid visualization of those sites can be instrumental for assessing the performance of the algorithms used. One of the most informative visualizations for a limited set of samples is the mismatchPlot (Fig. 1). It shows the coverage, mismatches and deleted bases for each sample in a genomic region and is generated directly from the tally data.

### 4.2 Mutation Spectrum Analysis

Mutation spectrum analysis compares frequencies of different types of mismatches across multiple samples (Alexandrov *et al.,* 2013) and can provide useful information regarding the mutation-generating mechanisms. **h5vc** offers the mutation Spectrum function to compute a mutation spectrum from a tally file (Supplementary Fig. S2). The mutation spectrum itself is a 4D-matrix with the layout (Sample, Prefix, Suffix, Mutation Type), from which the typical signatures of acting mutational processes can be extracted via non-negative matrix factorization using e. g. the R package NMF (Gaujoux and Seoighe, 2010).

## 5 CONCLUSION

Tallies stored in HDF5 files are a feasible and useful extension of the tool set of genome analysts. A concrete implementation of the tools necessary to make use of this data format is provided by the package **h5vc**. The associated documentation enables users to start using HDF5-based tallies immediately. Given the amount of genomics data that will have to be handled in the near future, this technology has the potential to become a valuable tool for genomic research.

## Supplementary Material

Supplementary Data
