# Supplementary material for: h5vc: scalable nucleotide tallies with HDF5
Source: Bioinformatics. 2014 Jan 21;30(10):1464–6. doi: 10.1093/bioinformatics/btu026 (PMC4016699; doi:10.1093/bioinformatics/btu026)
Supplement: Supplementary Data [file supp_30_10_1464__index.html]

h5vc: scalable nucleotide tallies with HDF5 — h5vc: scalable nucleotide tallies with HDF5 — Supplementary Data 

# h5vc: scalable nucleotide tallies with HDF5

## Supplementary Data

files

**Files in this Data Supplement:**

- Supplementary Data - pdf file
