# Supplementary material for: h5vc: scalable nucleotide tallies with HDF5
Source: Bioinformatics. 2014 Jan 21;30(10):1464–6. doi: 10.1093/bioinformatics/btu026 (PMC4016699; doi:10.1093/bioinformatics/btu026)
Supplement: Supplementary Data [file supp_btu026_draft.supplement_23DEC2013.pdf]

Dataset	Definition
Counts	table of observed mismatches with respect to the reference (4 dimensions) - `[bases x samples x strands x positions]`
Coverages	table of number of overlapping reads (3 dimensions) `[samples x strands x positions]`
Deletions	table of observed deletions of bases (3 dimensions) `[samples x strands x positions]`
Reference	one-dimensional vector containing the reference bases `[positions]`

Table 1. Overview of the datasets present in an HDF5 tally file giving definitions of their content and a representation of the dimensions of the datasets.

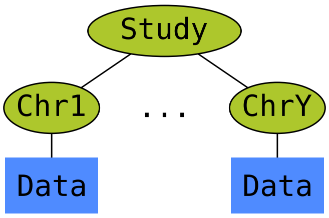

Supplementary Figure 1. Overview of the internal structure of a tally HDF5 file showing the tree structure with groups representing studies and chromosomes (contigs) as internal nodes and the 4 datasets defined in Supplementary Table 1 as leafs.

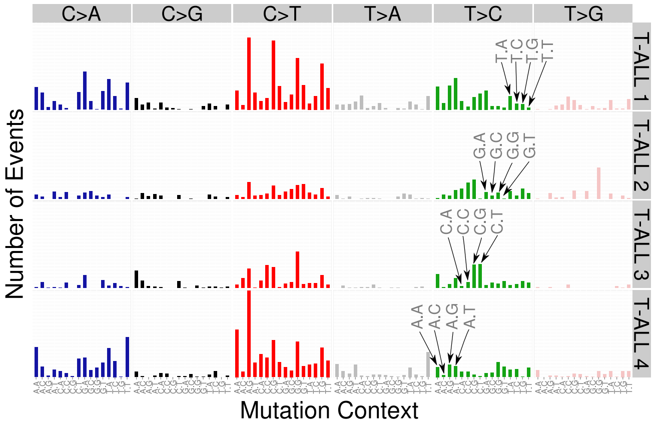

Supplementary Figure 2. Somatic mutation spectra plots, similar to those shown in Figure 2 of Alexandrov et al. (2013). The four rows of panels correspond to four cancer samples (T-ALL 1 to T-ALL 4), the six columns of panels to the possible base substitutions (`C>A`, `C>G`, `C>T`, `T>A`, `T>C`, `T>G`). Within each panel, a histogram of the mutations with that base substitution and all possible preceding and succeeding bases (i. e., the substitution's local sequence context) is shown, encoded as `Prefix.Suffix`, e.g `A.C`.

REFERENCES

Alexandrov LB, et al. Signatures of mutational processes in human cancer. *Nature*, 500(7463):415–421 (2013).
